# Differential Effects and Temporal Course of Attentional and Motivational Training on Excessive Drinking

**DOI:** 10.1037/pha0000038

**Published:** 2015-09-07

**Authors:** W. Miles Cox, Javad S. Fadardi, Steven G. Hosier, Emmanuel M. Pothos

**Affiliations:** 1 School of Psychology, Bangor University; 2 Department of Clinical Psychology, Ferdowsi University of Mashhad and School of Psychology, Bangor University; 3 School of Psychology, Bangor University; 4 Department of Psychology, City University London

**Keywords:** excessive drinking, attentional training, motivational training, alcohol abuse, brief interventions

## Abstract

Two cognitive-motivational variables that help to solidify drinkers’ intentions to drink are their alcohol attentional bias and their maladaptive motivation. The Alcohol Attention Control Training Programme (AACTP) was designed to rectify the former, and the Life Enhancement and Advancement Programme (LEAP) was designed to rectify the latter. The present study used a factorial design to compare the individual and combined effects of the 2 interventions on mean weekly drinking and atypical weekly drinking of 148 harmful drinkers (49% males, mean age = 28.8 years). A variety of other cognitive-motivational and demographic measures were also taken at baseline, and the drinking measures were reassessed at posttreatment and 3 and 6 months later. In comparison with LEAP, the effects of AACTP were less enduring. Combining AACTP and LEAP had few incremental benefits. These results suggest that AACTP would be more effective for achieving short-term reductions in drinking, whereas LEAP would be more effective for alleviating problematic drinking.

Excessive drinking is a serious problem in Western society with major psychological, social, and economic consequences. Although problem drinkers might recognize the negative consequences of their drinking and want to change, they often find it difficult to do so. Many variables contribute to a strong motivation to drink.

In an attempt to account for excessive drinking, Cox and Klinger (2011a) developed a motivational model of alcohol use, which brings together the biological, psychological, and sociocultural determinants of drinking into a unifying motivational framework. The model, originally published in 1988, has been widely cited (a few recent examples include Roos, Pearson, & Brown, 2015; Studer et al., 2014; Wardell & Read, 2014). The model has also been an impetus for a variety of research studies. Just one example is Cooper (1994), who developed a four-factor measure of drinking motives based on Cox and Klinger’s conceptual model. Cooper’s measure of drinking motives has been extensively used in research studies and has been widely cited. Basic research based on Cox and Klinger’s model has confirmed the importance of two major cognitive-motivational determinants of drinking. *First*, drinkers’ propensity to attend to alcohol-related stimuli and not to disengage their attention from these stimuli—referred to as alcohol attentional bias—reflects preoccupation with drinking. *Second*, drinkers’ maladaptive motivational structure prevents them from focusing on and successfully achieving healthy, adaptive goal pursuits as an alternative to drinking alcohol. Fadardi and Cox (2008) found, in fact, that alcohol attentional bias and motivational structure were the two significant predictors of excessive drinking that remained after a variety of other determinants of drinking had been controlled.

Previous research has shown that excessive drinkers and other substance abusers selectively attend to substance-related stimuli (Bruce & Jones, 2006; Klinger & Cox, 2011). Moreover, the degree of the attentional bias is proportional to the current level of substance use (Cox, Fadardi, & Pothos, 2006), and it is associated with users’ subjective craving (Field & Cox, 2008). Substance abusers also show greater attentional distraction for substance-related stimuli than they do for other goal-related stimuli (Cox, Blount, & Rozak, 2000; Fadardi, Ziaee, & Shamloo, 2009), which seems to reflect a lack of compelling, alternative incentives in their lives. Finally, Cox, Hogan, Kristian, and Race (2002) and Cox, Pothos, and Hosier (2007) found that alcohol abusers’ degree of attentional bias was a negative predictor of reductions in drinking 3 months later. Clearly, therefore, attentional bias is related in important ways to excessive drinking, and it appears to play a causal role in its development and maintenance (see Robinson & Berridge, 2003; Tiffany, 1990).

The Alcohol Attention-Control Training Programme (AACTP; Fadardi & Cox, 2009)—which is based on the alcohol Stroop task—is a computerized training technique for helping excessive drinkers overcome their automatic distraction for alcohol and thereby reduce their drinking. The alcohol Stroop task involves two categories of stimuli—alcohol-related (e.g., words such as *wine*, *beer*, or *tavern*) and emotionally neutral (e.g., words such as *table*, *door*, and *sidewalk*). Each word appears on a computer screen, typically in one of four colors (red, yellow, blue, or green). The participant’s task is to name as quickly and accurately as possible the color of the font in which the word appears, while ignoring the meaning of the word. Nevertheless, participants who have a concern about drinking alcohol are automatically distracted by the alcohol-related words, and they have slower RTs in naming them. The AACTP trains participants to ignore the task-irrelevant aspect of stimuli (their alcohol relatedness) and to respond progressively faster to the task-relevant aspect (the color). The training is designed to counteract the automatic cognitive processes leading up to drink-seeking and alcohol ingestion, by helping excessive drinkers gain better control over their alcohol attentional bias. In research to evaluate the AACTP, Fadardi and Cox (2009) found that participants who received the training showed reductions in both alcohol attentional bias and alcohol consumption, and the reductions were maintained at a 3-month follow-up.

Motivational structure refers to the kinds of goals people have and their ways of striving for them; some motivational patterns are more adaptive than others (Klinger & Cox, 2011). When, for example, people are able to strive for goals that they value and they are making progress in achieving them, they are more likely to feel that their life is meaningful, and they experience greater subjective well-being (see Klinger & Cox, 2011, p. 35). Such people are said to have an adaptive motivational structure. Adaptive motivation is also positively related to sense of control and intrinsic motivation (Shamloo & Cox, 2010) and to resilience (Fadardi, Azad, & Nemati, 2010). When, on the other hand, people’s responsivity to natural rewards is low or their pattern of goal-striving is maladaptive, they are more likely to drink alcohol or use other substances (Cox & Klinger, 2011a; Kalivas & Volkow, 2005; Murphy, Correia, Colby, & Vuchinich, 2005) and to experience alcohol-related problems (Cox et al., 2002; Hosier & Cox, 2011). Similarly, inverse relationships have been found between excessive drinkers’ having other satisfying incentives to enjoy and the ability to reduce their drinking (Tucker, Vuchinich, Black, & Rippens, 2006; Tucker, Vuchinich, & Rippens, 2002).

Drinkers’ motivational structure can be assessed with the Personal Aspirations and Concerns Inventory (PACI) or a related instrument (Cox & Klinger, 2011b). These instruments are idiothetic in the sense that respondents begin by providing idiographic descriptions of their current goals, which they then rate using nomothetic rating scales. On the PACI, respondents name their goal for achieving each of their major aspirations or for resolving each of their major concerns in various areas (e.g., career and employment, relationships, self-changes). They then rate each goal along various dimensions (e.g., commitment to goal attainment, degree of control over goal attainment, expected joy from goal attainment, expected chances of success). These ratings can be processed to provide indices and profiles that characterize the individual’s motivational structure.

From the information obtained from the PACI, Systematic Motivational Counseling (SMC; Cox & Klinger, 2011c) can be used to help substance abusers improve their maladaptive motivational patterns. The aim is to enable them to develop a fulfilling lifestyle that does not involve excessive use of alcohol or other drugs. SMC has been shown to reduce substance use, and the reduction is mediated by improvements in maladaptive motivation (e.g., Cox et al., 2003; Miranti & Heinemann, 2004). The current research advances the applicability of SMC by adapting the technique for use as a brief intervention with groups of participants. The new technique is called Life Enhancement and Advancement Programme (LEAP); it is described in detail in the Method section.

The major purpose of the current research was to assess the individual and combined effects of the AACTP and LEAP interventions on drinking behavior. One possibility is that the two kinds of interventions would operate independently of each other, so that the combined effects of the two kinds of training would be additive. Another possibility is that one of the interventions would moderate the effects of the other, which would be reflected as an interaction between the two kinds of training. A third possibility is that combining the two interventions would distract from the effectiveness of each when delivered alone.

Finally, we sought to evaluate how the effects of AACTP and LEAP training were manifested across time. One might expect, for example, that changing proximal determinants of drinking, which could be achieved using the AACTP, would be easier to accomplish than would effecting more fundamental changes in distal determinants, such as those that the LEAP targets. In this case, the effects of AACTP should be apparent earlier than those of LEAP. On the other hand, AACTP training used alone might be insufficient for resolving problems with excessive drinking, and the training effects might erode in time. If, however, the LEAP intervention can be used to help drinkers replace the function that alcohol serves with alternative incentives, one might expect less erosion of the effects of LEAP across time, compared with the effects of AACTP training. To assess these possibilities, the effects of AACTP and LEAP were measured at three time intervals after the baseline assessment—at posttreatment and 3- and 6-month follow-ups.

In summary, the present work advances previous research on attentional and motivational interventions in two important ways, by determining (a) the relative benefits of each intervention when it was delivered alone; and (b) whether combining the two interventions would produce additive, multiplication, or no additional benefits. We note that no intervention has previously been developed for examining the joint influence of cognitive and motivational variables in helping excessive drinkers to reduce their drinking.

## Method

### Participants

Participants were required to be 18 years old or older (the legal drinking age in the United Kingdom) and to have drunk above the U.K. Department of Health’s (2005) cut-off points for healthy drinking (i.e., 21 units of alcohol/week for males, 14 units/week for females; one unit = 10 ml of pure alcohol) for at least 1 week during the prior 12 weeks. They were recruited from the following sources: School of Psychology Community Participant Panel and Student Participant Panel (Bangor University, United Kingdom); community alcohol services (in North Wales); newspaper advertisements; posters and fliers displayed in general-practitioners’ waiting rooms and other public places; announcements posted on the Bangor University Intranet; and advertisements displayed on local buses. Bangor is a small city with a nonstudent population of approximately 18,575 and a student population of about 10,000. The city is situated on the North Wales coast in a predominantly rural area.

The recruitment announcement indicated that the purpose of the research was to teach heavy drinkers skills for reducing their drinking; hence, participation in the study was an indication of the drinkers’ motivation to change. This procedure was followed partly to obtain a more representative sample of the population of heavy drinkers and partly because of the difficulty of disambiguating a genuine desire to reduce drinking from an interest in, for example, taking part in the study for financial reward (which was intentionally kept at a modest level). The School of Psychology Ethics Committee approved the study. All participants gave informed, written consent, and they were paid a small cash amount for taking part in the study. The stated purpose of the payment was to help defray participants’ travel expenses. They were paid at the rate of £7 (approximately $10) per hour, or a total of £30 (approximately $45) for participating in all phases of the study.

### Measures

Participants were administered the following battery of tests:

#### Demographic characteristics

Participants’ demographic characteristics were measured with the Client Socio-Demographic and Service Receipt Inventory—European Version (CSSRI-EU; Chisholm et al., 2000). The CSSRI-EU asked participants about their age, sex, marital status, years of education, living situation (whether living alone or with a spouse or partner or with parents, other relatives, or nonrelatives), employment status (whether gainfully employed or engaged in voluntary or sheltered employment or unemployed), and first language (whether English, Welsh, or another language).

#### Alcohol consumption

Alcohol consumption was measured with the Drinking Record Questionnaire (DRQ, Fadardi, Cox, & Hogan, 2006), which asks about the quantity and frequency of participants’ typical and atypical drinking during the preceding 12 weeks. Both typical (usual) drinking and atypical (unusual) drinking were measured because the amount of alcohol that a person typically drinks can be quite different from the amount that the person drinks on atypical occasions. For social drinkers, atypical drinking is likely to mean drinking more than the person typically does. For excessive drinkers, however, atypical drinking might mean drinking less than the typical excessive amount. Measuring both kinds of drinking provides a more accurate appraisal of drinking patterns than measuring only typical drinking. Separately for weeks of typical and atypical drinking, participants indicated the type(s) of beverage(s) drunk, percentage of alcohol by volume (ABV%) in each beverage, and the quantity and frequency with which each was consumed. Two quantity-frequency indices of drinking were calculated: mean weekly quantity of alcohol consumed across the 12 weeks (i.e., mean weekly drinking; MWD) and mean quantity consumed during the atypical weeks (i.e., atypical weekly drinking; ATWD).

#### Alcohol-related problems

Problems associated with excessive drinking were measured with the Short Index of Problems (SIP; Forcehimes et al., 2007). The SIP yields a total score and scores on five subscales: physical, interpersonal, intrapersonal, impulse control, and social responsibility.

#### Motivation to change

The Readiness to Change Questionnaire (RTCQ; Heather, Rollnick, & Bell, 1993) was used to measure participants’ stated intentions to change their drinking during the next 3 months. RTC scores can be used to assign drinkers to one of three stages of change (precontemplation, contemplation, or action), and a total readiness-to-change score can also be derived.

#### Motivational structure

Participants’ motivational structure was measured with a computerized version of the Personal Aspirations and Concerns Inventory (PACI; Cox & Klinger, 2011b). On the PACI, respondents first name (or simply think about to themselves) their goal for achieving each of their aspirations or resolving each of their concerns. They then rate each goal using a variety of motivational scales, each of which ranges from 0 (*the least amount*) to 10 (*the greatest amount*). This baseline measure of motivational structure formed the basis for LEAP workshop sessions.

#### Satisfaction with life

Satisfaction with life was measured with the Satisfaction with Life Scale (SWLS; Diener, Emmons, Larsen, & Griffin, 1985). The SWLS is a 5-item instrument designed to measure the person’s global self-appraisal of his or her life.

#### Attentional bias

Participants’ attentional bias for alcohol and other goal-related stimuli was measured with the alcohol and goal-related computerized Stroop tasks similar to those used in previous studies (e.g., Fadardi & Cox, 2009). Alcohol, goal-related, and neutral words were used that were matched on word length and frequency and semantic relatedness. The goal-related words (e.g., health, friends, money, work) represented participants’ most frequently named personal goals in previous studies. Alcohol-interference and goal-interference scores were calculated, respectively, by subtracting each participant’s mean reaction time (RT) to the neutral stimuli from his or her mean RT to the alcohol-related or goal-related words. The alcohol interference scores were used to help participants set goals for reducing their alcohol attentional bias in the AACTP sessions.

### Interventions

#### Alcohol Attention-Control Training Programme

AACTP is a computerized training program that could be self-administered; however, in the present research, a research assistant (a doctoral-level student in psychology) administered the AACTP and guided each participant individually through the training.

The AACTP used three categories of stimuli. Two of the categories were presented individually on a computer monitor and comprised individual alcoholic or soft-drink bottles, each of which was surrounded by either a colored background or border in one of four colors—red, yellow, blue, or green. The participant’s task was to ignore the content of the pictures and name the surrounding color as quickly and accurately as possible by pressing a button on the keypad. In the third category, pairs of bottles appeared simultaneously on the screen. The participant was instructed to name the outline color of each nonalcoholic bottle as quickly as possible, while ignoring the alcoholic bottle.

Each of the four training sessions began with the single stimuli with colored backgrounds (easiest in the series); proceeded with the single stimuli with colored outlines (of intermediate difficulty); and continued with the paired stimuli (most difficult). After completing each session, the participant was given numerical and graphical feedback, including (a) number of errors and mean RT to the alcohol and nonalcohol stimuli; and (b) an interpretation of the results based on the person’s mean RTs, number of errors, and interference score. The goal was to motivate participants to become engaged in the training. Prior to each session, the participant was encouraged to set a goal for decreasing his or her errors and RTs to the colored dimension of the alcoholic bottles, relative to the nonalcoholic ones. The goal was for each participant to improve attentional control until his or her performance plateau had been reached. The plateau was defined as having RTs ≤ 1,000 ms, a near zero or negative interference score, and making fewer than 10% errors in each session.

All participants showed some progress in working through the training program; however, the experimenter’s emphasis was always on the progress that the person had made compared to his or her performance on the previous steps. Although all of the participants were capable of progressing through all the steps of the program, it might take some of them one or two extra exercises to reach their goal compared with others. However, we always avoided comparing the person with the performance of other participants. In other words, care was taken to insure that when the training sessions were terminated, participants felt good about their progress.

#### Life Enhancement and Advancement Programme

LEAP is a motivational intervention delivered in a workshop format in a group of five to 10 participants. The workshop leader does not have to be clinically trained; instead, the intervention is highly structured and consists of a series of exercises for participants to do. A training manual was developed for the leader to follow. In the present study, the leader was a research associate who had a Ph.D. in psychology.

The aim of the LEAP was to help excessive drinkers (a) understand how they had used alcohol to regulate their mood and affect and how doing so was related to their striving or not for goals in other life areas, (b) achieve their nondrinking goals more effectively, and (c) lead a satisfying life without using alcohol excessively.

The LEAP comprised four sessions. In the first session, participants were taught (a) how various factors related to people’s striving for goals—such as feeling a lack of control, not knowing steps to take, or having unattainable or unrealistic goals—can affect their satisfaction with life; and (b) how people’s lack of satisfaction in other areas of their lives can affect their motivation to drink alcohol. In the second session, they were asked to examine and discuss the goals that they had named on the PACI, which all participants had taken at the baseline assessment. In the third session, participants began completing a goal matrix to show how their goals helped or hindered one another. In doing so, they were helped to select valued, realistic, clearly defined goals to pursue that did not conflict with other goals. They also began constructing a goal ladder for achieving their selected goals, which included specifying actions to be taken and obstacles to be overcome. In the fourth session, they completed the goal ladder that had been started in the previous session. They were also asked to refine their goal selections and develop a plan for achieving long-term goals that would enhance their life satisfaction.

### Design

In a 2 × 2 design, the study crossed two levels of AACTP (present, absent) with two levels of LEAP (present, absent). The resulting four groups received only AACTP, only LEAP, both AACTP and LEAP, or neither AACTP nor LEAP. Simply measuring drinkers’ alcohol consumption can be regarded as an intervention in that self-monitoring triggered by the measurement leads to reductions in drinking (Miller & Wilbourne, 2002). The objective of the present design was to determine whether AACTP and LEAP would produce reductions in consumption over and above those produced by possible self-monitoring. This was the initial evaluation of the effectiveness of LEAP and of the combined LEAP and AACTP interventions.

### Procedure

At baseline, participants were administered the full assessment battery. A research assistant (a doctoral-level student in psychology) administered this assessment (and the subsequent assessments); she was aware of the group to which participants had been assigned. After completing it, they were randomly assigned to one of the four groups, but with the constraint (a) that the four groups were approximately equivalent at baseline in mean alcohol consumption, and (b) the groups were of approximately equal size. Participants in the AACTP and LEAP groups received a 1-hr session once a week for 4 weeks. Participants in the Combined Group received both a 1-hr AACTP session and a 1-hr LEAP session once a week for 4 weeks. It would, of course, have been impossible for the person delivering each of the interventions to be unaware of the group to which participants had been assigned.

Upon completion of his or her intervention, each participant was administered the measures of alcohol consumption and alcohol-related problems again. The postintervention assessment of the control participants was timed so that it occurred at the same interval following the baseline assessment as it did for the intervention groups. Three and 6 months after the postintervention assessment, participants in all four groups were again interviewed about their alcohol consumption and alcohol-related problems. Finally, participants in the control group were offered one of the interventions. All participants were provided with sources of help in the local community for alcohol-related problems, which they could contact should they feel that they needed to.

## Results

### Participants

Of the potential participants who volunteered, 148 (49% male) met the criteria for hazardous or harmful drinking and were included in the sample. All 148 participants completed the baseline assessment; 79.05% (48% male) of these (*n* = 117) returned for posttreatment assessment; of these, 73.50% (43% male) (*n* = 87) completed the first follow-up assessment; finally, of those who completed the first follow-up, 79.31% (43% male) (*n* = 69) returned for the second follow-up session. Thus, approximately 20% of participants dropped out at each assessment. Table 1 indicates the percentage of participants who dropped out of each group.[Table-anchor tbl1]

The mean age of the participants was 28.78 (*SD* = 14.38) years, and their mean education was 14.95 (*SD* = 3.06) years. Their posttreatment assessment occurred a mean of 35.49 (*SD* = 21.92) days after the baseline assessment, and the two follow-up assessments occurred a mean of 84.66 (*SD* = 33.39) days and 171.97 (*SD* = 57.64) days after the postintervention assessment. Participants had drunk excessively a mean of 8.42 (*SD* = 2.39) weeks during the preceding 12 weeks. Mean weekly alcohol consumption for the males was 75.16 (*SD* = 55.33) units; for the females, it was 44.05 (*SD* = 34.92) units. Thus, at baseline both the males and the females, on average, were drinking at a harmful level, according to the U.K. Department of Health’s criteria for harmful drinking (50 or more units of alcohol/week for males; 35 or more units of alcohol/week for females). On average, participants had drunk 9.55 (*SD* = 18.18) units of alcohol on their last drinking occasion, which had occurred an average of 1.71 (*SD* = 4.06) days prior to the baseline assessment.

Table 1 presents participants’ demographic characteristics, including their alcohol consumption at baseline. We also considered possible differences between males and females on other variables (measured at baseline) that were potentially relevant to excessive drinking and reductions in drinking: Readiness to change (RTC), alcohol-related problems (SIP), situational confidence (SCQ), positive affect (PA), negative affect (NA), satisfaction with life (SWL), and degree of alcohol dependence (LDQ). These results are presented in Table 2, which shows that the largest difference was in SWL, but when a Bonferroni-corrected threshold for significance (.05/7 = .007) was applied, this difference failed to reach significance.[Table-anchor tbl2]

### Drinking Reductions

Across the preintervention, postintervention, first follow-up (FU1), and second follow-up (FU2) assessments, participants’ alcohol consumption decreased significantly. MWD (units/week) was 59.39 (*SD* = 48.55), 43.21 (*SD* = 38.20), 35.03 (*SD* = 29.97), and 33.87 (*SD* = 30.68) at the four respective assessments, *F*(3, 219) = 24.49, *p* < .0005, $\eta^{2}=.25$. ATWD (units/week) was 13.84 (*SD* = 15.09), 10.01 (*SD* = 13.91), 6.85 (*SD* = 7.65), and 8.37 (*SD* = 13.98), respectively, for the four time points; *F*(3, 228) = 6.641, *p* < .0005, $\eta^{2}=.08$. Finally, it should be noted that at all four assessments participants’ ATWD was lower than their MWD, as would be expected given that participants were heavy drinkers. Although at baseline the two indices of drinking were significantly correlated with each other (*r*[148]) = .41, *p* < .0005, they shared only 17% of the variance, thus indicating that the two measures were largely independent. Because ATWD reflected a lower level of drinking, it might be expected that it would be less affected by the interventions than would MWD.

### Analysis

Despite the high attrition rate during the posttreatment phase of the study, missing values were not replaced. We retained all participants who completed the treatment phase of the study but who dropped out at later stages. We treated the corresponding data points as missing values in the analyses.

**Figure 1 fig1:**
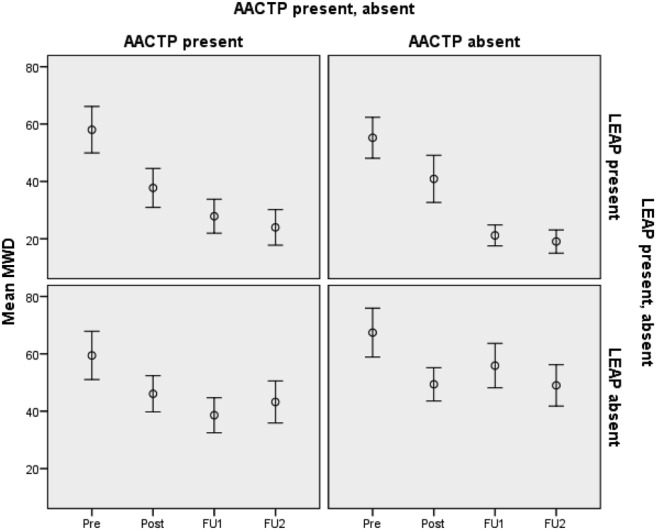
MWD at the four time points (baseline (pre), postintervention (post), FU1, and FU2). Error bars indicate one standard error of the mean.

The objective of the analysis was to determine the effectiveness of AACTP and LEAP in reducing participants’ alcohol consumption (MWD and ATWD). The individual and combined effects of the two interventions on changes from baseline in MWD at the three times points are shown in Figure 1. These results suggest that both AACTP and LEAP delivered individually reduced participants’ alcohol consumption but that little, if any, additional benefits accrued when the two interventions were combined.[Fig-anchor fig1]

A complication, however, is that the reduction in alcohol consumption shown in Figure 1 is partly due to the interventions but also partly due to factors that led participants to persevere with the study. We thus considered several variables on which the completers and noncompleters might have differed at baseline: RTC, SIP, SCQ, PA, NA, SWL, and LDQ. We used independent-samples *t*-tests to compare the completers and noncompleters on these variables (see Table 3). The results indicated that the completers and noncompleters differed on SIP, SWL, and LDQ, using a Bonferroni adjusted significance level of .05/7 = .007.[Table-anchor tbl3]

We carried out three mixed-design ANCOVAs to evaluate differential reductions in MWD depending on the group to which participants were assigned. In the first ANCOVA, the between-participants factors were AACTP (present, absent) and LEAP (present, absent); the within-participant factor was the time points in the study when MWD was measured (baseline and postintervention, in the first analysis), and the covariates were the SIP, SWL, and LDQ variables. In this first ANCOVA, effectiveness of the AACTP or LEAP interventions would be indicated by a two-way interaction between AACTP and LEAP. Evidence for the effectiveness of the combined intervention would be indicated by a three-way interaction. The remaining two ANCOVAs evaluating reductions in MWD were identical to the first one, except that in the first of these ANCOVAs, the within-participants factor included the baseline and FU1 time points, and in the second one, it included the baseline and FU2 time points. The reason why we conducted three separate ANCOVAs (one for each of the relevant time periods: pre-to-post; pre-to-FU1; pre-to-FU2) is that we had reason to expect that the impact of each of the interventions on MWD may manifest itself differently, across the different time periods (and this turned out to be the case).

The results, shown in Table 4, show that the benefits of AACTP and LEAP on MWD depend on when they are assessed. *First*, at the postintervention assessment, the effect of AACTP was marginally significant, but there was no effect for either LEAP or the combined intervention. *Second*, at the first follow-up, both interventions—AACTP, LEAP—were significant, but the combined intervention was not. *Third*, at the second follow-up assessment, the impact of LEAP on MWD remained highly significant, but the effect of AACTP was no longer significant, and the interaction between AACTP and LEAP was still not significant. These results suggest that AACTP is effective in producing short-term reductions in MWD, but that the reductions attenuate with time. The effects of LEAP, on the other hand, take longer to become manifest, but they are also more long lasting. Finally, when the two interventions were combined, there appear to have been no additional benefits over and above when either one of the interventions is delivered individually. This outcome is illustrated in Figure 1; for all relevant time points, there was no additional benefit of the combined intervention over and above what was achieved when AACTP or LEAP were delivered individually.[Table-anchor tbl4]

Sex was not included as an independent variable in the analyses because preliminary examination of the results (see Table 3) indicated that sex was not a moderating variable. This preliminary impression was confirmed by repeating the ANCOVAs described above, but this time with sex included as an additional independent variable. We were interested in seeing whether there were three-way interactions among the time-point factors, the interventions, and sex; an effect of sex on the combined intervention would be indicated by a four-way interaction. None of these interactions was significant (see Table 5).[Table-anchor tbl5]

**Figure 2 fig2:**
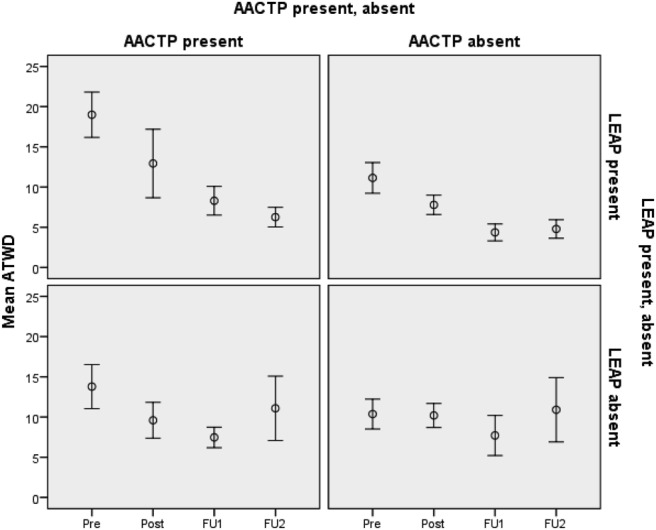
ATWD at the four time points (baseline (pre), postintervention (post), FU1, and FU2). Error bars indicate one standard error of the mean.

The statistical analyses evaluating reductions in ATWD were analogous to those evaluating reductions in MWD. Changes in ATWD across time as a function of the kind of intervention are shown in Figure 2; they reveal a picture similar to that for MWD. The results, shown in Table 6, indicate that when delivered individually, AACTP was significant at both the 3-month and 6-month follow-ups. On the other hand, as with MWD, the effects of LEAP on ATWD when delivered individually took longer to develop. The effect was significant at the 3-month follow-up assessment, and it was highly significant at the 6-month follow-up. Finally, the effects of the combined intervention were not significant at any of the time points. As with MWD, we also conducted the analyses by including sex as an independent variable. As with MWD, none of the relevant interactions was significant (see Table 7).[Fig-anchor fig2][Table-anchor tbl6][Table-anchor tbl7]

## Discussion

The major purpose of this study was to evaluate two promising interventions for excessive drinking—the Alcohol Attention Control Training Programme (Fadardi & Cox, 2009) and the Life Enhancement and Advancement Programme (LEAP)—a modified version of Systematic Motivational Counseling (SMC; Cox & Klinger, 2011c). Previous evaluations of the AACTP and of SMC have yielded positive results, but this was the first, preliminary evaluation of LEAP. Because AACTP and LEAP are designed to target different aspects of drinking behavior, it was important to directly compare the relative benefits of each one on drinking reductions and to determine whether combining the interventions would bring additional improvement. The present study, therefore, combined AACTP (presence, absence) and LEAP (presence, absence) in a factorial design. A variety of demographic and cognitive-motivational variables were measured at baseline, so that the impact of AACTP and of LEAP could be examined conservatively in relation to other putative determinants of changes in alcohol consumption. To determine how drinking reductions changed across time differentially for the AACTP and LEAP interventions, drinking-specific dependent variables (mean weekly drinking [MWD] and atypical weekly drinking [ATWD]) were measured immediately before and after each intervention and 3 and 6 months later.

We observed a large attrition rate of approximately 20% at each follow-up assessment point. Although this is undesirable, it is worth considering what alternative courses of action could have been adopted. One possibility would have been to incentivize participants for completing all phases of the study, for example, with a large monetary reward. However, such a procedure would mean that some participants would persevere with the study not because of their commitment to reduce their alcohol consumption, but because of the reward. We would not expect such participants to seriously engage with the interventions, and this would introduce noise with regard to the effectiveness of the interventions. In short, if these interventions were precursors of interventions that might be used in clinical practice, then dropout rates would be expected to be comparable with those of current treatments (Public Health England, 2013).

The dropout rate was approximately the same as Cox et al. (2007) obtained in a sample from the same geographical area and with similar demographic characteristics, although the 2007 study included only assessments and not an intervention. Nevertheless, it would have been inappropriate to artificially motivate participants to complete all of the sessions (e.g., by offering them a strong financial incentive) because doing so would have interfered with the intended function of the interventions. Moreover, we wanted to emulate naturalistic recruitment and retention conditions, that is, conditions that would provide a realistic estimate of the chances of being able to recruit and retain participants if the interventions were applied in clinical practice. Data from Public Health England (2013) recorded treatment dropout rates at 26%. Dropouts from treatment include people who leave a treatment program early because they feel that they have achieved all they wanted to by that point in time. Other patients simply disengage from treatment because they feel that they have made little or no progress.

Our main findings were that participants who received AACTP showed significant reductions in MWD, but only at the 3-month follow-up assessment (marginally significant reductions were observed at the postintervention assessment as well); significant effects for AACTP on ATWD were observed at the 3- and 6-month follow-ups. Beneficial effects of LEAP were observed on both MWD and ATWD. On MWD, the effect was significant at the 3-month follow-up, and was highly significant at the 6-month follow-up. On ATWD, the effect approached significance at the 3-month follow-up, and again was highly significant at the 6-month follow-up.

Two conclusions can be drawn about the relative effectiveness of AACTP and LEAP from this pattern of results. First, comparison of the effect sizes for changes in MWD across the 3- and 6-month follow-ups with changes in ATWD at the same time points suggests that changes in MWD were more substantial than those in ATWD. Thus, both of the interventions had a greater effect on reducing excessive drinking than on reducing drinking when it was at a moderate level. This outcome is intuitive, and a fruitful direction for future research would be to explore it in greater detail.

A second conclusion to be drawn from the current results is that the effects of AACTP and LEAP followed different temporal courses. AACTP had reduced MWD at the 3-month postintervention assessment, but the effect was no longer significant at the 6-month follow-up. The effects of AACTP on reductions in MWD appear, therefore, to attenuate with time. On the other hand, the significant effects of LEAP on both MWD and ATWD were maintained at the 6-month follow. It appears, therefore, that AACTP is sufficient to instigate rapid changes in the cognitive processes that are most proximal to decisions to drink, but that AACTP is relatively ineffective at consolidating these changes, so they do not have a lasting effect on drinking behavior. By contrast, the entrenched motivational patterns related to one’s everyday routine that the LEAP targets are difficult to change, but once in place the changes are relatively long-lasting. In short, these results again suggest that the AACTP would be more effective at bringing about short-term reductions in drinking, whereas the effects of LEAP would be more enduring.

There were no additional beneficial effects of combining AACTP and LEAP over effects achieved when each intervention was delivered individually. Perhaps the general lack of incremental effects was because of the manner in which delivery of the two interventions was timed. That is, participants who received both interventions received them simultaneously, and the dual exposure might have caused them to experience information overload (Klingberg, 2009; Soucek & Moser, 2010). Another possibility is that intervening at different levels concurrently (a cognitive and a motivational level) is difficult to accomplish, perhaps because of the effort required to achieve the changes that the interventions target. In retrospect, it would appear preferable for the two interventions to be delivered successively rather than simultaneously. The present results suggest that AACTP should be given prior to LEAP, because the beneficial effects of AACTP can be realized over a shorter period of time. Thus, it might be that the most effective approach to bring about drinking reductions would be to teach excessive drinkers first to disattend to alcohol stimuli before they are helped to address other issues that motivate them to drink. This possibility awaits confirmation in future research.

Another possibility for future research would be to compare the relative and combined effects of (a) cognitive bias modification other than attentional retraining, and (b) motivational interventions others than systematic motivational counseling or LEAP on reductions in alcohol consumption across time. These other techniques might include, for example, alcohol approach-avoidance training (e.g., Wiers et al., 2015) or motivational enhancement therapy (e.g., Dieperink et al., 2015). Finally, it is important for future research to establish the mechanisms through which cognitive bias modification and motivational counseling has beneficial effects on participants’ alcohol consumption. For instance, are reductions in drinking from cognitive bias modification mediated by reductions in alcohol attentional bias? Are reductions in drinking from motivational counseling mediated by improvements in motivational structure? For these questions to be answered, valid and reliable measures of alcohol attentional bias and motivational structure must be identified, but there currently is lack of consensus about what these measures are. Hopefully, future research will resolve this issue.

## Figures and Tables

**Table 1 tbl1:** Demographic Characteristics of the Four Groups of Participants

Intervention	*N*	Age (in years)	% Female	MWD units of alcohol at baseline	ATWD units of alcohol at baseline	% Dropouts
Control	29	26.4 (12.2)	31.0	67.4 (45.8)	10.4 (10.0)	55.2
AACTP	35	32.2 (15.8)	54.3	59.4 (49.8)	13.8 (16.2)	25.7
LEAP	42	30.0 (15.2)	57.1	55.2 (46.3)	11.1 (12.4)	54.8
AACTP & LEAP	42	26.5 (13.4)	54.8	58.0 (52.5)	19.0 (18.3)	61.9
*Note*. Cell entries for age, MWD, and ATWD indicate means and standard deviations. Dropouts indicate the percentage of participants who dropped out of the study at any point following the baseline assessment. One unit of alcohol is defined in the United Kingdom as 10 ml or 8 g of absolute alcohol.						

**Table 2 tbl2:** A Comparison of Males, Females on Variables on Potentially Relevant Variables

Measures	Females	Males	*T*	*df*	*p*
RTC	3.44	3.74	−.221	146	.825
SIP	11.68	13.33	−1.189	146	.236
SCQ	3.86	3.86	.003	146	.998
PA	33.03	32.92	.090	142	.928
NA	25.47	23.44	1.395	143	.165
SWL	21.57	18.52	2.481	145	.014
LDQ	14.67	17.05	−1.856	146	.066
*Note*. RTC measures readiness to change. SIP measures alcohol-related problems. SCQ measures situational confidence. PA measures positive affect. NA measures negative affect. SWL measures satisfaction with life. LDQ measures alcohol dependence. The columns *t*, *df*, and *p* report independent samples *t*-tests, between males, females, on the variable indicated on each row.					

**Table 3 tbl3:** A Comparison of Completers and Noncompleters on Variables on Which the Two Kinds of Participants Could Potentially Differ

Measures	Females	Males	*T*	*df*	*p*
RTC	2.47	4.70	−1.646	146	.102
SIP	10.61	14.38	−2.778	146	.006
SCQ	3.87	3.85	.163	146	.87
PA	33.27	32.68	.47	142	.639
NA	23.64	25.32	−1.146	143	.254
SWL	21.70	18.38	2.714	145	.007
LDQ	14.09	17.59	−2.757	146	.007
*Note*. RTC measures readiness to change. SIP measures alcohol-related problems. SCQ measures situational confidence. PA measures positive affect. NA measures negative affect. SWL measures satisfaction with life. LDQ measures alcohol dependence. The columns *t*, *df*, and *p* report independent samples *t*-tests, between completers, noncompleters, on the variable indicated on each row.					

**Table 4 tbl4:** ANCOVA Results Comparing the Interventions on Changes in Mean Weekly Drinking (MWD)

	AACTP			LEAP			AACTP & LEAP		
	*F*	*p*	η^2^	*F*	*p*	η^2^	*F*	*p*	η^2^
Pre-to-Post	3.449	.066	.030	1.768	.186	.016	.052	.820	.000
Pre-to-FU1	6.304	.014	.071	10.662	.002	.115	.114	.737	.001
Pre-to-FU2	1.883	.175	.027	13.895	<.0005	.172	1.810	.183	.026
*Note*. Covariates were scores from the Short Inventory of Problems, Satisfaction with Life scale, and Leeds Dependency Questionnaire. The *F* and *p* values are for the interaction between the factor for the indicated time points and the interventions. Evidence for combined effects of AACTP and LEAP would be indicated by a three-way interaction between the time-points factor and each of the two interventions. For the pre-to-post tests, degrees of freedom were 1, 110; for the pre-to-FU1 tests, they were 1, 82; and for the pre-to-FU2 tests, they were 1, 67. For pre-to-post, we had N(AACTP)=31, N(LEAP)=34, N(both)=29, N(neither)=24. For pre-to-FU1, we had N(AACTP)=28, N(LEAP)=25, N(both)=18, N(neither)=18. For pre-to-FU2, we had N(AACTP)=26, N(LEAP)=19, N(both)=16, N(neither)=13.									

**Table 5 tbl5:** ANCOVA Results Exploring Whether the Influence of the Interventions on Changes in Mean Weekly Drinking (MWD) Depends on Sex

	AACTP			LEAP			AACTP & LEAP		
	*F*	*p*	η^2^	*F*	*p*	η^2^	*F*	*p*	η^2^
Pre-to-Post	.204	.653	.002	1.175	.281	.011	.024	.878	.000
Pre-to-FU1	.076	.783	.001	1.059	.307	.013	.128	.722	.002
Pre-to-FU2	.005	.943	.000	1.186	.280	.018	.162	.689	.003
*Note*. Covariates were scores from the Short Inventory of Problems, Satisfaction with Life scale, and Leeds Dependency Questionnaire. The *F* and *p* values are for the interaction between the factor for the indicated time points, the interventions, and sex. For the pre-to-post tests, degrees of freedom were 1, 106; for the pre-to-FU1 tests, they were 1, 78; and for the pre-to-FU2 tests, they were 1, 63.									

**Table 6 tbl6:** ANCOVA Results Comparing the Interventions on Changes in Atypical Weekly Drinking (ATWD)

	AACTP			LEAP			AACTP + LEAP		
	*F*	*p*	η^2^	*F*	*p*	η^2^	*F*	*p*	η^2^
Pre-to-Post	1.116	.293	.010	.795	.374	.007	.001	.972	.0
Pre-to-FU1	6.641	.013	.071	3.890	.052	.044	.762	.385	.009
Pre-to-FU2	6.958	.010	.090	9.743	.003	.122	.196	.659	.003
*Note*. Covariates were scores from the Short Inventory of Problems, Satisfaction with Life scale, and Leeds Dependency Questionnaire. The *F* and *p* values are for the interaction between the factor for the indicated time points and the interventions. Evidence for combined effects of AACTP and LEAP would be indicated by a three-way interaction between the different time-point factor and each of the two interventions. For the pre-to-post tests, degrees of freedom were 1, 113; for the pre-to-FU1 tests, they were 1, 85; and for the pre-to-FU2 tests, they were 1, 70. For pre-to-post, we had N(AACTP)=32, N(LEAP)=34, N(both)=29, N(neither)=26. For pre-to-FU1, we had N(AACTP)=29, N(LEAP)=25, N(both)=20, N(neither)=18. For pre-to-FU2, we had N(AACTP)=26, N(LEAP)=19, N(both)=18, N(neither)=14.									

**Table 7 tbl7:** ANCOVA Results Exploring Whether the Influence of the Interventions on Changes in Atypical Weekly Drinking (ATWD) Depends on Sex

	AACTP			LEAP			AACTP + LEAP		
	*F*	*p*	η^2^	*F*	*p*	η^2^	*F*	*p*	η^2^
Pre-to-Post	.320	.573	.003	2.146	.146	.019	.127	.722	.001
Pre-to-FU1	.889	.348	.011	.195	.660	.002	.108	.743	.001
Pre-to-FU2	.024	.877	.000	.454	.503	.007	2.772	.101	.040
*Note*. Covariates were scores from the Short Inventory of Problems, Satisfaction with Life scale, and Leeds Dependency Questionnaire. The *F* and *p* values are for the interaction between the factor for the indicated time points, the interventions, and sex. For the pre-to-post tests, degrees of freedom were 1, 109; for the pre-to-FU1 tests, they were 1, 81; and for the pre-to-FU2 tests, they were 1, 66.
